# Efficacy and safety of carbetocin applied as an intravenous bolus compared to as a short-infusion for caesarean section: study protocol for a randomised controlled trial

**DOI:** 10.1186/s13063-016-1285-5

**Published:** 2016-03-22

**Authors:** Salome Dell-Kuster, Irene Hoesli, Olav Lapaire, Esther Seeberger, Luzius A. Steiner, Heiner C. Bucher, Thierry Girard

**Affiliations:** Department of Anaesthesiology, Surgical Intensive Care, Prehospital Emergency Medicine and Pain Therapy, University Hospital Basel, 4031 Basel, Switzerland; Basel Institute for Clinical Epidemiology and Biostatistics, Basel, Switzerland; Department of Obstetrics and Antenatal Care, University Hospital Basel, Basel, Switzerland; Department of Clinical Research, University Hospital Basel, Basel, Switzerland

**Keywords:** Carbetocin, Mode of administration, Caesarean section, Uterine tone, Haemodynamic stability

## Abstract

**Background:**

The two most commonly used uterotonic drugs in caesarean section are oxytocin and carbetocin, a synthetic oxytocin analogue. Carbetocin has a longer half-life when compared to oxytocin, resulting in a reduced use of additional uterotonics. Oxytocin is known to cause fewer cardiovascular side effects when administered as a short-infusion compared to as an intravenous bolus. Based on these findings, we aim at comparing carbetocin 100 mcg given as a slow intravenous bolus with carbetocin 100 mcg applied as a short-infusion in 100 ml 0.9 % sodium chloride in women undergoing a planned or unplanned caesarean delivery. We hypothesise uterine contraction not to be inferior to a bolus application (primary efficacy endpoint) and greater haemodynamic stability to be achieved after a short-infusion than after a bolus administration, as measured by heart rate and mean arterial blood pressure (primary safety endpoint).

**Methods/Design:**

This is a prospective, double-blind, randomised controlled, investigator-initiated, non-inferiority trial taking place at the University Hospital Basel, Switzerland.

Uterine tone is quantified by manual palpation by the obstetrician using a linear analogue scale from 0 to 100 at 2, 3, 5 and 10 minutes after cord clamping. We will evaluate whether the lower limit of the confidence interval for the difference of the maximal uterine tone within the first 5 minutes after cord clamping between both groups does not include the pre-specified non-inferiority limit of −10. Both haemodynamic secondary endpoints will be analysed using a linear regression model, adjusting for the baseline value and the dosage of vasoactive drug given between cord clamping and 1 minute thereafter, in order to investigate superiority of a short-infusion as compared to a bolus application.

We will follow the extension of CONSORT guidelines for reporting the results of non-inferiority trials.

**Discussion:**

Haemodynamic stability and adequate uterine tone are important outcomes in caesarean sections. The results of this trial may be used to optimise these factors and thereby increase patient safety due to a reduction in cardiovascular side effects.

**Trial registration:**

Clinicaltrials.gov NCT02221531 on 19 August 2014 and www.kofam.ch SNCTP000001197 on 15 November 2014.

## Background

Uterotonic drugs are routinely used after caesarean section (CS) to reduce blood loss and the risk of postpartum haemorrhage (PPH) [1]. PPH is defined as a blood loss of at least 500 ml after vaginal birth and at least 1000 ml after CS and/or the need for blood transfusion within 24 hours after delivery [2, 3] and represents the most important cause of maternal morbidity [4]. As PPH is more common after CS than after vaginal delivery [5] and as the rate of CS is rising, the incidence of PPH is expected to increase accordingly.

The two most commonly used uterotonic drugs after operative delivery are oxytocin and carbetocin, a synthetic oxytocin analogue. Carbetocin has some advantages over oxytocin due its four- to ten-times longer half-life [6], allowing for a single injection. Moreover, a recent meta-analysis showed that carbetocin significantly reduces the use of additional uterotonics as compared to oxytocin, although the risk of severe PPH remains similar [7].

Carbetocin is associated with cardiovascular side effects including hypotension and a compensatory increase in heart rate similar to those of oxytocin [8, 9]. According to randomised trials with oxytocin, the extent of these cardiovascular side effects seems to be dependent on dose [10, 11] and the rate of administration [12]. The manufacturer of oxytocin requires the drug to be given as a short-infusion to maintain cardiovascular stability. In contrast, the manufacturer of carbetocin recommends a bolus injection of carbetocin. Whether carbetocin administration as short-infusion offers the optimal balance between contractile benefit to minimise maternal haemorrhage and reduce cardiovascular side effects is unclear.

In this randomised controlled trial, we aim to investigate whether carbetocin 100 mcg applied as a short-infusion in 100 ml 0.9 % sodium chloride as compared to a bolus injection minimises the cardiovascular side effects (first secondary safety endpoint) without compromising uterine tone (primary efficacy endpoint) in women undergoing a planned or unplanned caesarean delivery.

## Methods/Design

### Study design and setting

This is a prospective, single-centre, randomised, double-dummy, investigator-initiated, non-inferiority trial conducted at the University Hospital Basel, Switzerland. Study recruitment commenced in December 2014 and is expected to last for 16 months. The study was approved by the regional Ethics Committee (Ethikkommission Nordwestschweiz (EKNZ), EKNZ 2014–088, 13 March 2014). The study was registered in a national and an international trial registry (www.kofam.ch SNCTP000001197 and clinicaltrials.gov NCT02221531).

### Randomisation and blinding

Randomisation for a 1:1 allocation of the study treatment is provided through an online-randomiser to ensure allocation concealment. This online-randomiser was integrated into the password-protected database. The randomisation took place immediately before the CS after having controlled the inclusion and exclusion criteria. The randomisation list was generated in Intercooled Stata Version 13.1 (StataCorp, College Station, TX, USA) using variable block sizes between 2 and 8. The list is safely stored in a separate and sealed file, which will only be opened if unblinding becomes necessary. This is considered to be unlikely since the concomitant treatment in case of insufficient uterine tone is independent of the mode of administration of carbetocin.

**Fig. 1:**
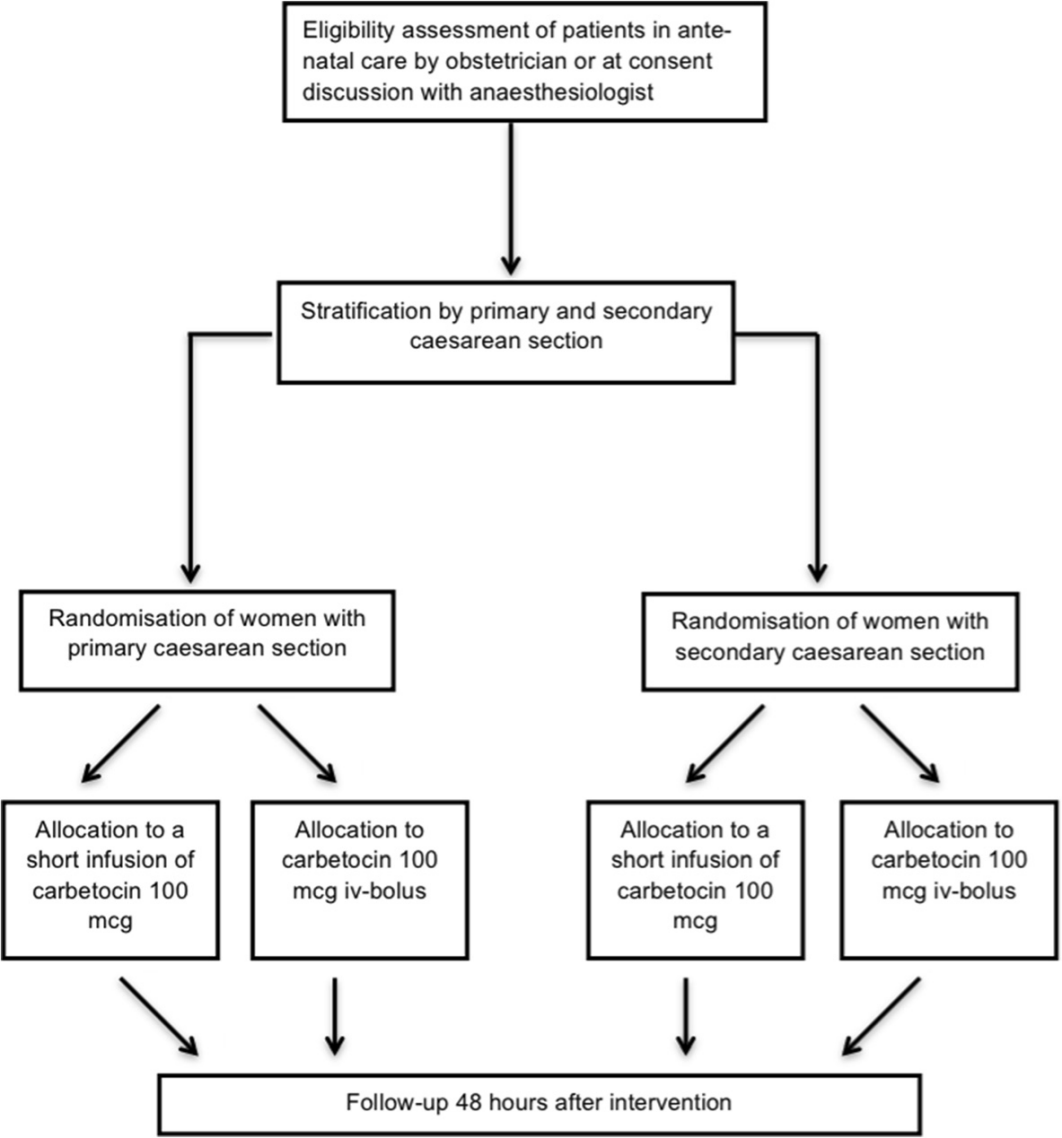
Trial flow chart showing trial procedure of recruitment, stratified randomisation and follow-up visit

Subjects are randomised on arrival in the operating theatre to minimise the risk of dropouts after randomisation. To achieve similar numbers, randomisation is stratified for planned or unplanned CS (Fig. 1).

Two members of the study team (ES and SDK) ensure correct administration of the study drug, transfer of the haemodynamic parameters, and collect intraoperative data, neither of whom is involved in the patients’ care. The study nurse (ES) always prepares the study drug. Correspondingly, patients, all care providers (obstetric and anaesthesia team) as well as the statistician (SDK) will remain blinded to group allocation until the data are analysed.

### Selection and withdrawal of participants

#### Recruitment

Participants are identified at their pre-anaesthesia visit and by screening the daily list of patients scheduled for a CS (Fig. 1). The study staff or the anaesthetist in charge informs the patients about all aspects pertaining to participation in the trial and invites them to participate.

#### Informed consent

Eligibility is routinely assessed by the anaesthesia or obstetric team in order to decide whether the patient is competent to provide consent to participate in the study. Written informed consent will be obtained from all participants. Participants are clearly informed that trial participation is voluntary and that they are free to withdraw at any time without effect on subsequent care. Recruitment of study participants by all members of the research team is in line with the guidance for Good Clinical Practice for obtaining consent [13].

#### Inclusion criteria

Healthy women with a singleton pregnancy undergoing a planned or unplanned CS after at least 37 completed weeks of gestation under regional anaesthesia (spinal, epidural or combined spinal-epidural anaesthesia) aged older than 18 years and with signed informed consent are eligible.

#### Exclusion criteria

The following exclusion criteria are being applied: (1) emergency CS, (2) unplanned CS due to fetal distress, (3) maternal comorbidities such as severe cardiovascular disorders including (pre-)eclampsia, kidney or liver disorders, coagulopathies, as well as epilepsy, (4) uterine malformation, (5) fetal malformation, (6) known hypersensitivity to carbetocin or oxytocin, and (7) the inability to read and understand the participant’s information.

### Study procedure

#### Standard care

After arrival of the patient in the operating theatre, the patient is positioned with a left uterine tilt to avoid aorto-caval compression. A fluid co-load of 500 ml hydroxyethyl starch HES 6 % is infused while the patient is monitored with pulse oximeter, electrocardiogram and non-invasive blood pressure cuff around the upper arm. Spinal anaesthesia by single-shot technique using hyperbaric bupivacaine 0.5 % 10 mg, fentanyl 10 mcg and morphine 100 mcg is applied in right lateral position. If an epidural catheter is already in place, 15–20 ml lidocaine 2 % with carbon dioxide and adrenaline 1:200,000 are administered through the catheter after an intrathecal or intravasal displacement has been excluded. After the application of the spinal anaesthesia, the patient is positioned in a left-tilted recumbent position. The CS is performed according to the in-house standard procedure applying the Misgav Ladach method for the laparotomy [14]. Following uterine incision, delivery of the baby and cord clamping, the placenta is delivered by cord traction or manually, if necessary.

#### Study treatment in intervention and control group

After cord clamping, each patient will receive a 1-ml bolus and a short-infusion of 100 ml 0.9 % sodium chloride, one of which contains of carbetocin 100 mcg and the other of placebo (double-dummy technique). The study intervention only contains the mode of administration of carbetocin. An independent study nurse prepares the study medication (1-ml bolus and 100-ml short-infusion with or without carbetocin 100 mcg). The nurse is the only involved person with access to the randomisation results. All syringes and short-infusions will be labelled according to the Good Manufacturing Practice Guidelines with the study number (1–140) being the only differentiating feature between the study drugs.

#### Concomitant treatments

In case of insufficient uterine tone, the patient will be treated according to the current standard of care. There are six options: (1) uterine massage, (2) sulprostone 4–8 mcg/min, (3) 600 mcg misoprostol rectal, (4) oxytocin infusion 20–40 U/2 h, (5) Bakri™ balloon, and (6) surgical (including Tachosil®) or radiological (embolisation) intervention. The choice of one or several of these interventions is according to the severity of uterine atony and at the discretion of the treating obstetrician.

#### Laboratory analyses

Haemoglobin and haematocrit levels are measured preoperatively and 2 days postoperatively in order to estimate the perioperative blood loss taking into account the calculated pregnancy blood volume and the percent blood volume lost [15, 16].

#### Follow-up visit

All participants have a follow-up visit 2 days postoperatively to rule out side effects of carbetocin and to ensure that no further problems have arisen.

#### Data monitoring and stopping rules

The Ethics Committee agreed that monitoring would be performed by the interdisciplinary study team. This team will evaluate the progress of the trial, verify the accuracy and completeness of the case report forms (CRFs), and ensure that all protocol requirements and investigator’s obligations are being fulfilled. The progress of the trial is evaluated every second month. The overall distribution of uterine tone will be assessed after 2 and 8 months in a blinded way, i.e. without considering group allocation. Extreme values are investigated further by reviewing the intraoperative course and by determining reasons for low uterine tone.

### Study objectives

#### Primary (efficacy) endpoint

The primary efficacy endpoint of this trial is the maximum uterine tone within the first 5 minutes after cord clamping assessed by manual palpation, and rated on a linear analogue scale (LAS) from 0 (completely atonic) to 100 (fully contracted), by the obstetrician.

#### First secondary endpoint (or primary safety endpoint)

The haemodynamic parameters (lowest mean arterial pressure and highest heart rate within the first 5 minutes after administration of the study drug) are considered as primary safety endpoints.

#### Further secondary endpoints

- Mean change over time of uterine tone measured 2, 3, 5 and 10 minutes after cord clamping
- Median dose of as well as number of patients requiring vasoactive drugs during CS: phenylephrine, ephedrine, atropine, nitroglycerine during the time period before cord clamping, between cord clamping until 1 minute after the end of the short-infusion, and between the second measurement and the end of surgery
- Mean estimated blood loss within the first 48 hours calculated by multiplying the calculated maternal blood volume by the percent of blood volume lost [15]
- Need for additional uterotonics (type and amount) during or immediately after surgery
- Tolerability assessment intraoperatively and 2 days postoperatively by monitoring the following side effects of the study drug: ST segment analysis to detect signs of myocardial ischaemia, angina pectoris, facial flushing, headache, nausea and vomiting
- Administration of any blood product (i.e. packed red cells, fresh frozen plasma) or coagulation factors such as tranexamic acid or fibrinogen during or immediately after surgery
- Median time and type of placenta removal, i.e. by cord traction or manually

#### Measurement of uterine tone

The obstetrician provides an assessment of the uterine tone by manual palpation using a LAS ranging from 0 to 100. Manual palpation of the uterus is the standard used to assess uterine tone in daily clinical practice.

#### Haemodynamic measurements

Blood pressure is measured non-invasively using a cuff on the upper arm. All haemodynamic data (including ST segment alterations) are recorded and stored in the anaesthesia monitor (IntelliVue MX800, Philips AG Healthcare, Zurich, Switzerland). The programme ixTrend from ixellence (ixellence GmbH, Wildau, Germany) is used to ensure direct transfer from the monitor to the study computer.

### Statistics

#### Sample size justification

To be 80 % sure that the lower limit of a one-sided 97.5 % confidence interval (CI) will be above the non-inferiority limit of −10 points on the LAS, 63 patients per group are required assuming a standard deviation (SD) of 20. Offsetting a maximal dropout of 10 %, this leads to a total sample size of 140 patients. The non-inferiority margin has been defined to be −10 on the LAS by the four most senior obstetricians at our institution. The SD had been derived from the results of a trial by Butwick et al., who used the same scale to assess the uterine tone [11].

#### Data management

All data from patients will be stored anonymously. Each patient will be assigned a unique patient-identity (ID) consisting of a three-digit number. The patient’s ID, name and date of birth will be stored separately in a different file in order to be able to examine queries in case of implausible values (reversible anonymisation). Only members of the study team have access to the data.

An electronic database featuring web-based access and using a secure (Secure Sockets Layer: SSL) connection is used for data entry. The database is password-protected and all data are encrypted. A consistency check is performed by logical data testing on the browser and on a random sample of 5 % by an independent study member.

#### Statistical analysis plan

The following baseline characteristics will be used to investigate comparability of both trial arms: age, body mass index, parity, gravidity, gestational week, risk factors for PPH, number of previous CS, planned or unplanned CS, reason for CS, experience level of the obstetrician, batch number. Continuous variables will be described by using the mean (SD) or median (interquartile range) as appropriate, categorical variables by using count (percentage).

In order to answer the primary research question, a CI of the mean difference in LAS score of the maximal uterine tone within the first 5 minutes after cord clamping will be calculated. If the lower limit of this CI does not include the pre-specified non-inferiority limit of −10, an inferiority of a short-infusion of more than 10 points on the LAS as compared to a bolus application is unlikely. In order to improve efficiency, an additional model will be built adjusting for the most relevant confounders, such as experience of the obstetrician, parity or number of previous CS, number of risk factors for PPH (multiple pregnancy, abnormal placentation, history of PPH or uterine atony), duration of surgery, and duration of antenatal oxytocin infusion. In order to compare the change of the uterine tone over time between both study groups, a generalised estimation equation (GEE) [17] will be built using an exchangeable correlation matrix (with an interaction term between treatment group and time). Moreover, the uterine tone over time will be displayed graphically.

The haemodynamic secondary outcomes (lowest mean arterial blood pressure and highest heart rate within the first 5 minutes after the administration of the study drug) will be analysed using a linear regression model adjusting for the corresponding baseline value and the amount of vasoactive drug administered during this period. In a further analysis, the course over time of mean arterial pressure and heart rate will be modelled using a GEE model with an exchangeable correlation matrix, using a knot at the average time of the lowest mean arterial pressure adjusted for the dosage of vasopressors administered, in order to compare the trend over time in both groups. Moreover, the change over time in blood pressure and heart rate will be displayed graphically. The incidence of any clinically relevant ST segment changes in both groups will be compared using a chi-squared or a Fisher’s exact test as appropriate. Further secondary endpoints will only be analysed descriptively. The comparison of the calculated blood loss will be adjusted in a linear regression model for whether or not the mother is breastfeeding.

All analyses and graphs will be performed using Intercooled Stata Version 13.1 for Mac (StataCorp, College Station, TX, USA). We will follow the Consolidated Standards of Reporting Trials (CONSORT) statement and its extension for non-inferiority trials for the future report [18, 19].

#### Definition of dataset analysed

A per-protocol and an intention-to-treat analysis will be performed, and only if both approaches support non-inferiority will the trial be considered as supporting our non-inferiority hypothesis.

### Reporting of adverse events

Participants are asked about any side effect including (serious) adverse events immediately and 2 days postoperatively. All these events are recorded and monitored until the patient has completely recovered or until no causal relationship to the study drug administration has been established. All serious adverse events will be reported to the Ethics Committee and the sponsor within the appropriate time frame.

The trial will be terminated early in the event of relevant safety concerns that threaten the study participants in any way. Therefore, we will monitor the incidence of PPH in a blinded manner and stop the study early for safety concerns if the incidence rate should rise above the average value of about 5 % PPH in CS [20].

## Discussion

This randomised controlled investigator-initiated trial prospectively evaluates how the uterine contractility and haemodynamic parameters are affected by the mode of administration of carbetocin in patients undergoing a CS. Based on the findings of reduction in cardiovascular side-effects with a short-infusion as compared to a bolus injection found for oxytocin [12], our study hypothesis is that a slower administration rate of carbetocin (using a short-infusion) minimises cardiovascular side effects without compromising uterine tone. In the following, we discuss considerations regarding trial design and chosen endpoints.

### Choice of non-inferiority design

The aim of this trial is to reach greater haemodynamic stability using the slower administration method (primary safety endpoint) while ensuring non-inferiority with regard to the uterine tone (primary efficacy endpoint) and ultimately the risk of elevated blood loss. Since superiority in haemodynamic endpoints can be achieved with fewer patients, we decided to choose as our primary endpoint non-inferiority in uterine tone.

### Choice of primary endpoint

We consider the choice of the appropriate measurement of our primary endpoint as a main challenge in this trial. The choice of our primary outcome is based on a randomised controlled trial performed by Butwick et al. [11] who also used a LAS ranging from 0 to 10 to assess uterine tone in a similar setting. We expanded the scale from 0 to 100 to avoid the fact that some outcome assessors used decimal places (e.g. 7.5) while others did not. Moreover, manual palpation of the uterus represents the standard method to assess uterine tone in daily clinical practice. To reduce bias, especially when using a subjective primary outcome measurement, we have established and will maintain blinding by using a double-dummy technique for study drug administration in patients and all care providers until the statistical analysis has been performed. We have chosen to abstain from a binary assessment of the uterine tone as it would be too parallel to our secondary outcome variable ‘any additional uterotonics – yes/no’. The objective efficacy outcome of interest would have been intraoperative blood loss. Blood loss during PPH is often estimated visually, but visual assessment has been shown to be imprecise, usually underestimating blood loss particularly concerning larger volumes [21–24]. We have, therefore, decided to choose the uterine tone as our primary outcome variable. We consider blood loss as a secondary endpoint, calculated by taking into account the pre- and postoperative haematocrit and the estimated blood volume of the parturient [15].

### Haemodynamic measurement method

We chose haemodynamic stability, defined as lowest mean arterial pressure and highest heart rate within the first 5 minutes after the administration of the carbetocin. After evaluating the options for continuous invasive and non-invasive blood pressure measurements, we have decided to measure blood pressure non-invasively using a cuff on the upper arm. We have considered the accuracy and the precision of the continuous non-invasive methods insufficient to justify their use in the context of this trial. Although the risk for complications of an intra-arterial catheter is low [25], the potentially severe side effects do not justify invasive blood pressure monitoring.

This sample size allows showing a superiority of the short-infusion over the bolus administration in mean arterial blood pressure by at least 5 mmHg with 80 % power and a type-1 error of 5 % assuming a SD of 10. Thus, even a clinically irrelevant difference in efficacy could be found with our data. However, we need to acknowledge that we are not able to show a minor difference in the incidence of serious drops in blood pressure with our sample size.

### Choice of study population

This trial will include patients undergoing a planned and an unplanned CS. Patients undergoing a unplanned CS often have already received oxytocin to induce or enhance labour, thus, showing a different response to the intraoperative dosage of carbetocin. Therefore, we are using a stratified randomisation for planned and unplanned CS. Excluding these patients would limit the generalisability of our study results.

### Recruitment

**Fig. 2:**
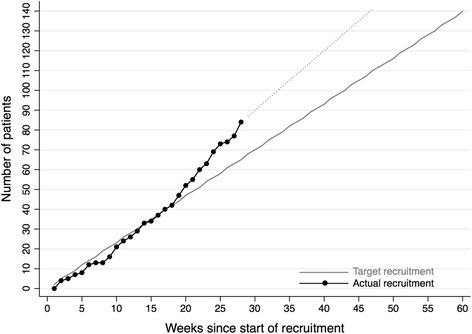
Target and actual recruitment rate to achieve the given sample size within the planned recruitment period

Poor recruitment is the most frequently reported cause for discontinuation of a clinical trial [26]. However, due to good interdisciplinary cooperation, adequate personal and financial resources and a high acceptance for clinical research, we are able to exceed the target recruitment rate (Fig. 2). Additionally, an amendment was handed in shortly after the beginning of the recruitment to incorporate patients with a breech position fetus. Within the same amendment, we have also translated the study information and the consent into English to be able to address more patients.

### Implications for future clinical practice

Haemodynamic stability and adequate uterine contractility are important outcomes in CS. The results of this trial may be used to optimise these factors and thereby increase safety in parturients due to reduced cardiovascular side effects.

## Trial status

The trial is currently enrolling patients. Upon submission of this manuscript, 60 % of the patients have been enrolled. The average enrolment rate is three (range one to seven) patients every week.

## Abbreviations

CI: confidence interval
CONSORT: Consolidated Standards of Reporting Trials
CS: caesarean section
GEE: generalised estimation equation
LAS: linear analogue scale
PPH: postpartum haemorrhage
SD: standard deviation
